# Remodeling of O Antigen in Mucoid Pseudomonas aeruginosa via Transcriptional Repression of *wzz2*

**DOI:** 10.1128/mBio.02914-18

**Published:** 2019-02-19

**Authors:** Ashley R. Cross, Joanna B. Goldberg

**Affiliations:** a Department of Pediatrics, Division of Pulmonary, Allergy and Immunology, Cystic Fibrosis, and Sleep, Emory University School of Medicine, Atlanta, Georgia, USA; b Emory+Children’s Center for Cystic Fibrosis and Airway Disease Research, Emory University School of Medicine, Atlanta, Georgia, USA; University of Washington; Harvard Medical School; Ohio State University

**Keywords:** *Pseudomonas aeruginosa*, alginate, lipopolysaccharide

## Abstract

Pseudomonas aeruginosa is an opportunistic pathogen that causes chronic lung infections in people with cystic fibrosis (CF). Chronic P. aeruginosa isolates generally do not express O antigen and often have a mucoid phenotype, which is characterized by the overproduction of the exopolysaccharide alginate. Therefore, O antigen expression and the mucoid phenotype may be coordinately regulated upon chronic adaption to the CF lung. Here we demonstrate that PDO300, a mucoid strain derived from the nonmucoid laboratory isolate PAO1, does not produce very long O antigen due to decreased expression of Wzz2, the very long O antigen chain length control protein, and that mucoid clinical isolates express reduced levels of Wzz2 compared to nonmucoid isolates. Further, we show that forcing the expression of very long O antigen by PDO300, by providing *wzz2* in *trans*, does not alter alginate production, suggesting that sugar precursors are not limited between the two biosynthesis pathways. Moreover, we confirm that AmrZ, a transcription factor highly expressed in mucoid strains, is a negative regulator of *wzz2* promoter activity and very long O antigen expression. These experiments identify the first transcriptional regulator of O antigen chain length in P. aeruginosa and support a model where transition to a chronic mucoid phenotype is correlated with downregulation of very long O antigen through decreased Wzz2 production.

## Abstract (precis)

Detection of mucoid Pseudomonas aeruginosa, characterized by the overproduction of alginate, is correlated with the establishment of a chronic pulmonary infection and disease progression in people with cystic fibrosis (CF). In addition to the overproduction of alginate, loss of O antigen lipopolysaccharide production is also selected for in chronic infection isolates. In this study, we have identified the regulatory network that inversely regulates O antigen and alginate production. Understanding the regulation of these chronic phenotypes will elucidate mechanisms that are important for the establishment of a long-term P. aeruginosa lung infection and ultimately provide an opportunity for intervention. Preventing P. aeruginosa from chronically adapting to the CF lung environment could provide a better outcome for people who are infected.

## INTRODUCTION

Pseudomonas aeruginosa is capable of causing chronic lung infections in people with cystic fibrosis (CF) (1, 2). Upward of 50% of people with CF are infected with P. aeruginosa, and lifelong infections are the leading cause of morbidity and mortality (3–6). CF is caused by mutations in the cystic fibrosis transmembrane conductance regulator (*cftr*) that result in altered ion transport and improper lung function (7, 8). This altered lung environment increases mucus accumulation in the lungs, reducing mucociliary clearance, which ultimately leads to bacterial persistence (9). Attempts at treating these life-threatening infections have fallen short due to P. aeruginosa’s ability to adapt to the CF lung and survive in the altered lung environment (10). This survival is reflected by the expression of rare phenotypes that distinguish chronic CF isolates from P. aeruginosa obtained from other sources or types of infections. The accumulation of these features is often referred to as the “chronic infection phenotype” (11).

One distinct chronic phenotype that has been observed is related to the expression of lipopolysaccharide (LPS). Environmental and acute infection isolates express an LPS-smooth phenotype, while P. aeruginosa isolates from chronic pulmonary infections are often LPS-rough, meaning they do not express O antigen (11–16). In LPS-smooth strains, the serotype-specific O antigen expressed is characterized based on size; long O antigen is regulated by the chain length control protein Wzz1, while very long O antigen is regulated by the chain length control protein Wzz2 (17–20).

In addition to the loss of O antigen expression, P. aeruginosa strains isolated from chronic infections are often mucoid (21, 22). Furthermore, these mucoid clinical strains are LPS-rough (23, 24). Detection of the mucoid phenotype, characterized by the overproduction of the exopolysaccharide alginate, is correlated with pulmonary disease progression (25–28). The most common mutations that lead to mucoid conversion in CF isolates are found in *mucA* (21, 29–31). MucA is an anti-sigma factor responsible for sequestering AlgT, the sigma factor that initiates transcription of the alginate biosynthesis operon and approximately 300 other genes of the AlgT regulon (32). When MucA is inactivated, AlgT constitutively transcribes the alginate biosynthesis operon, resulting in overproduction of alginate and the mucoid phenotype.

Given the apparent correlation between alginate overexpression and loss of O antigen in chronic infection isolates, it has been speculated that alginate and O antigen are coordinately controlled. In support of this premise, Kelly et al. compared the LPS profiles of the nonmucoid laboratory strain PAO1 and a series of phage-induced mucoid variants (33). They noted that the mucoid strains had lost expression of the high-molecular-weight portion of the LPS molecule and that nonmucoid reversion could restore production. Ma et al. (34) expanded upon the results of Kelly et al. by comparing O antigen production of PAO1 to that of PDO300, a well-studied isogenic mucoid variant of PAO1 (35). PDO300 contains the most common clinically observed *mucA* mutation *mucA22* (21). The authors observed that high-molecular-weight O antigen was reduced in PDO300 compared to PAO1. Both groups suggested that the overproduction of multiple mannose-rich exopolysaccharides results in a competition for a shared sugar pool by O antigen and alginate biosynthesis pathways. However, these results do not account for why the low-molecular-weight fractions of O antigen are unaffected since both low- and high-molecular-weight O antigens contain the same sugars.

Previous research has primarily focused on the mucoid phenotype and the regulation of alginate biosynthesis. In contrast, it is not known how O antigen is regulated during chronic infections, even though loss of O antigen expression is a common chronic phenotype. Given the failure of the shared precursor model to adequately explain why nonmucoid reversion of clinical isolates does not restore O antigen production or why only one chain length of O antigen is affected by mucoid conversion, we fill this gap in knowledge by, instead, identifying an overlapping transcriptional regulator that inversely controls alginate and O antigen expression at a genetic level. Understanding the coordinated regulation between the mucoid phenotype and O antigen production will elucidate mechanisms that are selected for during the establishment of a long-term infection and inform us about host-pathogen interactions during chronic infections. We suggest that interfering with the expression of these chronic infection phenotypes and the subsequent adaption of P. aeruginosa to the CF lung environment could provide a better outcome for people living with CF.

## RESULTS

### Mucoid strains produce less Wzz2 than nonmucoid strains, resulting in less very long O antigen.

**FIG 1:**
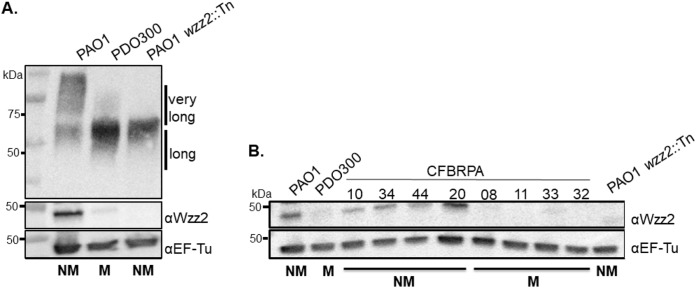
Mucoid strains produce less Wzz2 than nonmucoid strains, resulting in less very long O antigen. (A) Analysis of protein and LPS extracts from nonmucoid and mucoid strains by Western blotting. Wzz2 or EF-Tu (loading control) was visualized using anti-Wzz2 or anti-EF-Tu antibodies. Serotype O5-specific antibodies were used to visualize O antigen production. PAO1 *wzz2*::Tn is a transposon mutant from the PAO1 library (68, 69) and is used as a control for no Wzz2 or very long O antigen production. (B) CF isolates CFBRPA10, 34, 44, and 20 are nonmucoid, while CF isolates CFBRPA08, 11, 33, and 32 are mucoid. These isolates were obtained from the Emory-Children’s Healthcare Cystic Fibrosis Biospecimen Registry (CFBR). All Western blots are representative of three or more independent experiments. Abbreviations: NM, nonmucoid; M, mucoid; Tn, transposon.

Previous studies by Kelly et al. and Ma et al. showed that mucoid strains often do not express high-molecular-weight O antigen (33, 34). To begin to understand the mechanism responsible for the change in O chain length expression in mucoid strains, we first sought to confirm the observation made by Ma et al. (34). We isolated LPS and compared the O antigen profiles between nonmucoid (NM) PAO1 and mucoid (M) derivative PDO300 (35). When PAO1 LPS was separated based on size and probed using serotype O5-specific antigen antibodies, long and very long O antigen modalities were clearly visible (Fig. 1A). When LPS isolated from PDO300 was separated in the same manner, little high-molecular-weight O antigen was observed (Fig. 1A) as previously reported (34). We specifically noted that the O antigen modality of PDO300 looks similar to that of a PAO1 *wzz2*::Tn transposon mutant (19). This transposon is inserted in the first half of *wzz2*, likely disrupting Wzz2 protein function. Wzz2 is known to regulate the very long O antigen lengths, and in the absence of *wzz2*, PAO1 does not make very long O antigen (Fig. 1A).

We therefore hypothesized that loss of this high-molecular-weight O antigen is the specific loss of very long O antigen chain lengths in PDO300 and further that this is likely due to decreased production of Wzz2. Using polyclonal antibody to Wzz2 in Western blot analysis of protein extracts from PAO1, PDO300, and PAO1 *wzz2*::Tn, Wzz2 production of each strain was examined; reactivity was seen with an ∼49-kDa protein, as expected. Reactivity was also consistently seen with a protein of ∼65 kDa in all of the samples tested, including the *wzz2* mutant. Because of this, we are confident this cross-reacting band does not belong to Wzz2, and it has been cropped from the images. In support of our hypothesis, Wzz2 production was reduced in PDO300 compared to PAO1 (Fig. 1A). Comparison of protein levels by densitometry analysis determined that PDO300 makes about 65% less Wzz2 than PAO1. PAO1 *wzz2*::Tn, as expected, does not make Wzz2 (Fig. 1A). Altogether, these data indicate that the loss of high-molecular-weight O antigen expression in PDO300 is due to decreased expression of Wzz2 and thereby loss of very long O antigen modalities.

As Wzz2 is conserved among P. aeruginosa strains (19), we also compared Wzz2 and O antigen production in serotype O10 laboratory strain PA14 and a mucoid derivative that we generated (PA14 *mucA22*). PA14 *mucA22* has reduced levels of Wzz2 and fewer high-molecular-weight O antigen chain lengths than PA14 (see Fig. S1 in the supplemental material), confirming that the inverse relationship between alginate production and O antigen chain length is not strain or serotype specific.

10.1128/mBio.02914-18.3 FIG S1 Mucoid PA14 has reduced levels of Wzz2 and fewer very long O antigen chain lengths than nonmucoid PA14. Analysis of Wzz2 and serotype O10 antigen production. Samples were prepared and Western blotting was performed as described for Fig. 1. Abbreviations: NM, nonmucoid; M, mucoid. Download FIG S1, PDF file, 0.5 MB. Copyright © 2019 Cross and Goldberg. 2019 Cross and Goldberg This content is distributed under the terms of the Creative Commons Attribution 4.0 International license.

Since loss of O antigen expression is a recognized phenotype of CF isolates, we next determined whether decreased expression of Wzz2 by PDO300 held true in mucoid P. aeruginosa CF isolates. We used the Wzz2 polyclonal antibody to screen a series of random nonmucoid and mucoid CF isolates obtained from the Cystic Fibrosis Biospecimen Registry (CFBR). CFBRPA10, 34, 44, and 20 are all nonmucoid strains and, overall, expressed higher levels of Wzz2 than the mucoid isolates CFBRPA08, 11, 33, and 32 (Fig. 1B). Wzz2 was barely detectable in all of the mucoid isolates, and these appeared similar to the PAO1 *wzz2*::Tn control. CF isolates are generally LPS-rough and nontypeable (13); thus, O antigen production was not monitored in these strains.

### Overexpression of *wzz2* in PDO300 increases very long O antigen production.

**FIG 2:**
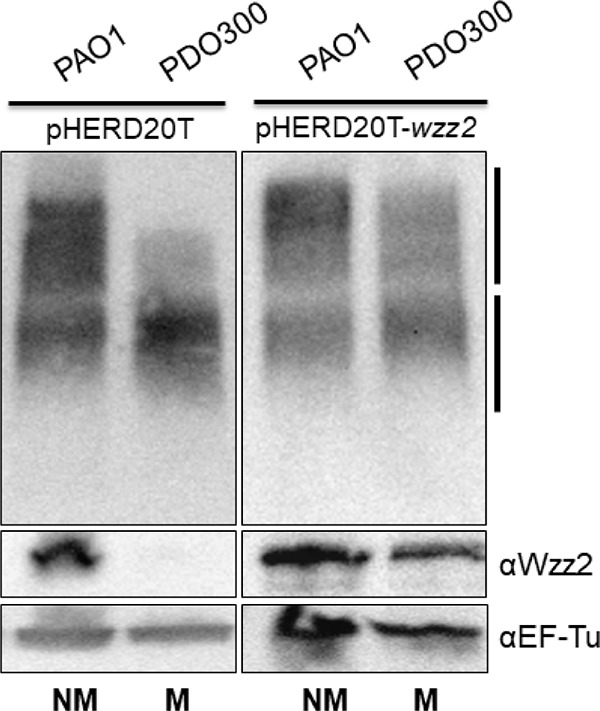
Overexpression of *wzz2* in PDO300 increases very long O antigen production. O antigen and Wzz2 production of strain overexpressing *wzz2* on pHERD20T (right) compared to vector-only control (left). All strains were grown in 0.4% l-arabinose inducer. Western blotting was performed as described for Fig. 1. Abbreviations: NM, nonmucoid; M, mucoid.

We next wanted to determine if we could restore expression of Wzz2 and very long O antigen to PDO300 by providing *wzz2* in *trans* and whether this would alter alginate production. If there is a shared pool of precursor sugars, we should not be able to express very long O antigen in PDO300 without compromising alginate production. To test this, we cloned *wzz2* behind an arabinose-inducible promoter contained on the plasmid pHERD20T (36) to generate pHERD20T-*wzz2*. This plasmid has a P. aeruginosa origin of replication and can be maintained in multicopy to facilitate overexpression of genes. pHERD20T-*wzz2* was transferred into PAO1 and PDO300, and production of Wzz2 and very long O antigen was monitored when *wzz2* was induced using 0.4% l-arabinose and compared to a vector-only (pHERD20T) control. When *wzz2* was overexpressed in PAO1, which already expresses very long O antigen, there was a modest increase in the amount of very long O antigen produced (Fig. 2). When *wzz2* was overexpressed in PDO300, we successfully restored Wzz2 and very long O antigen production to PAO1 levels (Fig. 2). There was no effect on Wzz2 or very long O antigen production when PAO1 pHERD20T and PDO300 pHERD20T vector-only controls were grown in 0.4% l-arabinose.

To determine if alginate production had been impacted in PDO300 when *wzz2* and very long O antigen were overexpressed, we monitored the mucoid phenotype. When grown on a plate containing 0.4% l-arabinose to induce *wzz2* expression, PDO300 pHERD20T-*wzz2* maintained a mucoid phenotype (data not shown). We quantified the amount of alginate that was made by PDO300, a PDO300 vector-only control (PDO300 pHERD20T), and PDO300 pHERD20T-*wzz2* when each strain was grown to stationary phase in the absence or presence of the inducer. PDO300 pHERD20T-*wzz2* grown without inducer produced 570.52 µg/ml of alginate (Table 1). When grown in inducer, PDO300 pHERD20T-*wzz2* had a 12% decrease in alginate production. As a control, we also compared alginate expression of the vector-only PDO300 control and noted a 21% decrease in alginate production when this strain was grown with inducer compared to growth without inducer (Table 1). Altogether, this suggests that there is no major difference in alginate produced when very long O antigen is made by a mucoid strain, suggesting that a limited pool of sugar precursors, under these conditions, does not account for loss of very long O antigen production in PDO300. This supports our hypothesis that decreased very long O antigen in PDO300 results from decreased expression of Wzz2 rather than a competition of sugar precursors.

**TABLE 1:** Alginate produced by strains overexpressing *wzz2*

Strain	Inducer (0.4% l-arabinose)	Alginate (µg/ml)	SD	Fold change
PDO300	−	615.89	75.94	
	+	610.50	126.59	0.99
PDO300 pHERD20T	−	706.99	142.33	
	+	555.00	210.39	0.79
PDO300 pHERD20T-*wzz2*	−	570.52	213.50	
	+	503.03	181.18	0.88

### The *wzz2* steady-state mRNA levels and promoter activity are repressed in PDO300.

**FIG 3:**
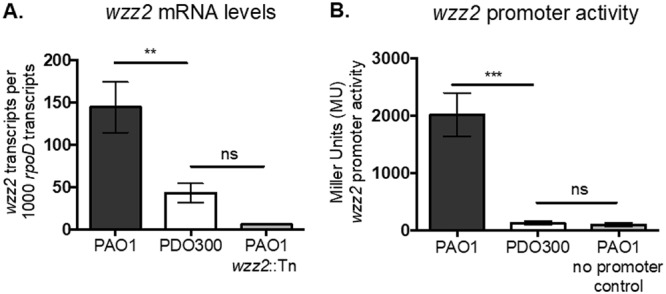
The *wzz2* steady-state mRNA levels and promoter activity are repressed in PDO300. (A) qRT-PCR to examine mRNA expression in PAO1 compared to PDO300. qPCR was performed using primers specific to *wzz2* and normalized to the housekeeping gene *rpoD*, which is known to be stably expressed (37). The PAO1 *wzz2* transposon mutant (PAO1 *wzz2*::Tn) was used as a negative control. (B) *wzz2* was fused to a promoterless *lacZ* containing an optimized ribosome-binding site (RBS) to make a transcriptional fusion. This fusion was used to measure *wzz2* promoter activity in each strain using β-galactosidase assays. Significance for each experiment was determined using one-way ANOVA with Tukey’s multiple-comparison analysis. Error bars represent SD from three biological replicates with technical triplicates. **, *P* < 0.01; ***, *P* < 0.001; ns, not significant.

To determine how Wzz2 is regulated in PDO300, we investigated both *wzz2* steady-state mRNA levels and transcription initiation. We utilized quantitative reverse transcriptase PCR with *wzz2* gene-specific primers, which anneal on sites upstream and downstream of the transposon insertion, to measure relative *wzz2* transcript levels in PAO1, PDO300, and control strain PAO1 *wzz2*::Tn, which does not express *wzz2* (Fig. 3A). Transcript levels were normalized to the housekeeping gene *rpoD* (37). PDO300 expressed about one-third the relative number of *wzz2* transcripts compared to PAO1 (Fig. 3A). Since *wzz2* mRNA expression is reduced in PDO300, we suspected that *wzz2* is transcriptionally repressed in this strain. To test this, we fused the 5′ promoter region of *wzz2* to a promoterless *lacZ* and inserted this construct, in single copy, into the CTX phage attachment site in PAO1 and PDO300. Subsequently, β-galactosidase enzymatic activity was used as a readout of *wzz2* promoter activity in each strain. As speculated, analysis of *wzz2* promoter activity revealed that PDO300 had a significant 17-fold decrease in *wzz2* promoter activation compared to PAO1 (Fig. 3B), indicating that *wzz2* is transcriptionally repressed in PDO300.

### Nonmucoid isolates of mucoid strains express more Wzz2 and very long O antigen.

**FIG 4:**
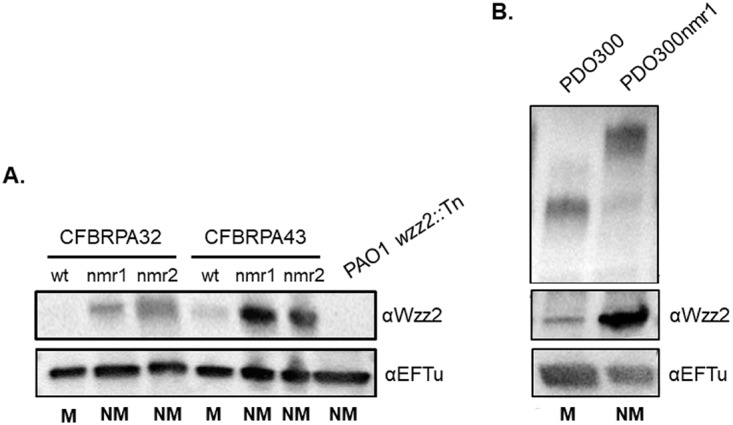
Nonmucoid isolates of mucoid strains express more Wzz2 and very long O antigen. (A) Nonmucoid revertants of mucoid CF isolates were isolated by daily passage in the laboratory. (B) PDO300nmr1 is a nonmucoid revertant of PDO300. Western blotting was performed as described for Fig. 1. Abbreviations: NM, nonmucoid; M, mucoid.

To further interrogate the relationship between Wzz2 and alginate production, we monitored Wzz2 expression in nonmucoid revertants of two mucoid CF isolates (38). The mucoid phenotype is unstable, and these strains frequently revert to nonmucoid in the laboratory (39). We hypothesized that loss of alginate production by a mucoid strain would restore Wzz2 expression. Importantly, isolation of nonmucoid revertants also allows us to study the effect of alginate and Wzz2 regulation in isogenic clinical isolates. We used Western blot analysis to compare Wzz2 production levels in mucoid clinical isolates CFBRPA32 and CFBRPA43 as well as two isogenic nonmucoid revertants of each. Each pair of nonmucoid revertants was obtained from the same mucoid parental culture. Both pairs of nonmucoid revertants, nmr1 and nmr2, had substantial increases in Wzz2 expression compared to each isogenic mucoid parental strain (Fig. 4A).

Frequently, nonmucoid reversion results from inactivating mutations in the sigma factor *algT* (30, 31, 40, 41). We sequenced *algT* in each nonmucoid revertant and discovered that three of the four strains contained mutations in the *algT* coding sequence. CFBRPA32nmr1 and nmr2 both had an in-frame duplication of bp 127 to 135 (GACGCCCAG). This results in insertion of amino acids DAQ. Since the two revertants had the same mutation, this is likely a result of a single clone overtaking the entire population early in passaging of this culture. CFBRPA43nmr1 had no mutations identified in the *algT* coding sequence, and therefore, a mutation upstream in the regulatory region or elsewhere in the chromosome resulted in nonmucoid reversion. Finally, CFBRPA43nmr2 had a C-to-A transversion at nucleotide 245 of *algT*, resulting in a threonine-to-asparagine change at amino acid 82. Both amino acids are polar and uncharged. These data provide a connection between *algT* and the regulation of Wzz2.

Since clinical isolates are often LPS-rough (23, 24), whether nonmucoid reversion is sufficient to restore very long O antigen expression remained unclear. Therefore, we utilized PDO300, which can express O antigen, to determine if nonmucoid reversion would restore very long O antigen production. We isolated a nonmucoid revertant of PDO300, PDO300nmr1, by daily serial passaging in a static broth culture. Interestingly, sequencing of *algT* in this nonmucoid revertant revealed the same duplication of bp 127 to 135 in *algT* as described for the CFBRPA32 nonmucoid revertants. We isolated and separated LPS by SDS-PAGE and analyzed O antigen expression using serotype-specific antibodies. As predicted, nonmucoid reversion of PDO300 restored very long O antigen production and resulted in increased Wzz2 production compared to PDO300 (Fig. 4B).

### Overexpression of *algT* results in less very long O antigen and decreased *wzz2* promoter activity.

**FIG 5:**
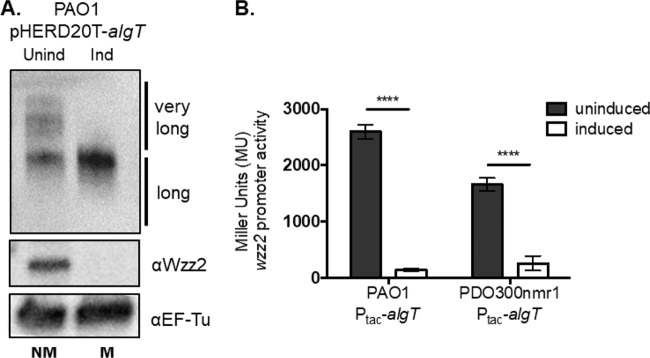
Overexpression of *algT* results in less very long O antigen and decreased *wzz2* promoter activity. (A) Wzz2 and O antigen were monitored when *algT* coding was overexpressed using pHERD20T. Uninduced (Unind) expression indicates growth without inducer, and induced (Ind) indicates growth in 0.4% l-arabinose. Western blotting was performed as described for Fig. 1. Abbreviations: NM, nonmucoid; M, mucoid. (B) *wzz2* promoter activity was monitored when *algT* was uninduced or induced using 1 mM IPTG. β-Galactosidase assays were performed as described for Fig. 3B. Error bars represent SD from three biological replicates with technical triplicates. Significance was determined using two-way ANOVA with Sidak’s multiple-comparison analysis. ****, *P* < 0.0001.

To expand upon our observations that mutations in *algT* result in increased Wzz2, we next sought to determine if overexpression of *algT* in *trans* would decrease Wzz2 and very long O antigen. To do this, we overexpressed *algT* on multicopy plasmid pHERD20T in PAO1, which usually does not express large amounts of *algT*. We then compared the effects of uninduced and induced pHERD20T-*algT* in PAO1 on Wzz2 and O antigen production. PAO1 grown without inducer and therefore with low *algT* expression produced both Wzz2 and very long O antigen. In contrast, when *algT* was induced with 0.4% arabinose, PAO1 produced no Wzz2 or very long O antigen and looked strikingly similar to PDO300 (Fig. 5A).

We next investigated whether overexpression of *algT* would repress *wzz2* promoter activity. To test this, we cloned *algT* behind an IPTG-inducible promoter in a mini-Tn*7*T plasmid and inserted this construct in single copy into PAO1 and PDO300nmr1 at the Tn*7* transposon insertion sites. When *algT* is overexpressed, nonmucoid strains become mucoid (41, 42). We measured the amount of alginate produced by PAO1 and PDO300nmr1 when *algT* was induced compared to when it was uninduced. PAO1 became mucoid when grown on inducer and produced a large 161.85-fold-increase in alginate compared to when *algT* was uninduced (Table S1). In contrast, when we quantified alginate production from PDO300nmr1 when *algT* was overexpressed, the mucoid phenotype was slightly restored. However, there was no significant increase in the amount of alginate that was made compared to that under uninduced conditions (Table S1). Since AlgT is known to regulate its own transcription in a positive-feedback loop (41, 43), we suspect that disrupting this autoregulation and only supplying *algT* in single copy fails to promote alginate overexpression. It could also be possible that this particular mutation in *algT* results in a dominant negative phenotype inactivating AlgT.

10.1128/mBio.02914-18.5 TABLE S1 Alginate produced by strains overexpressing *algT*. Download Table S1, PDF file, 0.1 MB. Copyright © 2019 Cross and Goldberg. 2019 Cross and Goldberg This content is distributed under the terms of the Creative Commons Attribution 4.0 International license.

To measure *wzz2* promoter activity when *algT* was overexpressed, we inserted the *wzz2* promoter-*lacZ* fusion at the CTX site of PAO1 and PDO300nmr1. Both strains contain an IPTG-inducible copy of *algT* at the Tn*7* site. First, we measured *wzz2* promoter activity in PAO1 when *algT* was overexpressed. When *algT* was induced, PAO1 had a significant 18-fold decrease in *wzz2* promoter activity compared to when *algT* was uninduced (Fig. 5B). We also measured *wzz2* promoter activity in PDO300nmr1 grown without inducer. When *algT* was uninduced, PDO300nmr1 had high levels of *wzz2* promoter activity (Fig. 5B). When PDO300nmr1 was grown on inducer to express *algT*, *wzz2* promoter activity was reduced 6-fold. Taken together, these data pinpoint AlgT as the critical inverse regulator of alginate production and very long O antigen.

### Overexpression of *amrZ* represses *wzz2* promoter activity.

**FIG 6:**
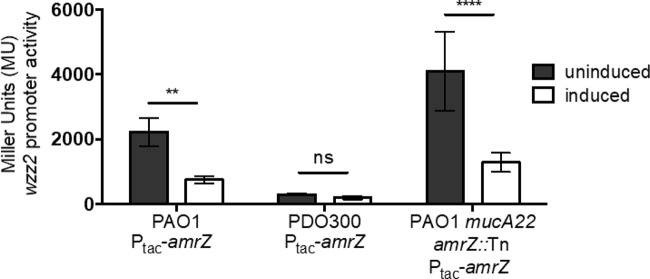
Overexpression of *amrZ* represses *wzz2* promoter activity. *wzz2* promoter activity was monitored when *amrZ* expression was uninduced or induced using 1 mM IPTG. β-Galactosidase assays were performed as described for Fig. 3B. The *mucA22* allele was inserted into PAO1 *amrZ*::Tn, a transposon mutant from the PAO1 library (68, 69), to generate PAO1 *mucA22 amrZ*::Tn. Error bars represent SD from three biological replicates with technical triplicates. Significance was determined using two-way ANOVA with Sidak’s multiple-comparison analysis. **, *P* < 0.01; ****, *P* < 0.0001; ns, not significant.

When active, AlgT transcribes the genes encoding three global transcriptional regulators: AlgB, AlgR, and AmrZ (44–47). We reasoned that one of these regulators would be a likely candidate for regulating *wzz2* transcription. Published microarray data for AlgB and AlgR did not provide evidence that *wzz2* was part of either regulon under the conditions tested (48–50). On the other hand, published RNA-sequencing and ChIP-sequencing data by Jones et al. provided evidence that, when *amrZ* is overexpressed in PAO1, AmrZ will bind upstream of *wzz2* and reduce *wzz2* mRNA production (51). The predicted binding site of AmrZ is CGATAGCATAATG at −88 to −75 nucleotides upstream of the *wzz2* start codon (51). In order to directly test whether AmrZ regulates *wzz2* transcription initiation, we reproduced an experiment similar to the one performed by Jones et al. by overexpressing *amrZ* in PAO1 and then measuring *wzz2* promoter activity. To do this, we inserted the *amrZ* coding sequence downstream of an IPTG-inducible promoter and inserted this construct at the Tn*7* attachment site of PAO1. This strain (PAO1 P_tac_-*amrZ*) also contains the *wzz2* promoter-*lacZ* fusion at the CTX site so that we can measure *wzz2* promoter activity when *amrZ* is being overexpressed. When *amrZ* expression is induced in the PAO1 background, this strain background had 4-fold less *wzz2* promoter activity than under uninduced conditions (Fig. 6). This was comparable to *wzz2* promoter levels in the PDO300 background (PDO300 P_tac_-*amrZ*), which were not altered when *amrZ* was induced or uninduced.

Likewise, to determine if disruption of *amrZ*, in the context of a mucoid strain, would alleviate repression of *wzz2,* we inserted the *mucA22* allele from PDO300 into a PAO1 *amrZ* transposon mutant to generate PAO1 *mucA22 amrZ*::Tn. Inserting *mucA22* into PAO1 replicates the original construction of PDO300. We then assayed *wzz2* promoter activity in PAO1 *mucA22 amrZ*::Tn by inserting the *wzz2* promoter-*lacZ* fusion at the CTX site of this strain. Supporting our premise, when *amrZ* was inactivated and grown under uninduced conditions, PAO1 *mucA22 amrZ*::Tn had about a 14-fold increase in *wzz2* promoter activity over PDO300 (Fig. 6). We then complemented the *amrZ* defect by providing *amrZ* in *trans*. When *amrZ* was overexpressed in PAO1 *mucA22 amrZ*::Tn, *wzz2* promoter activity was reduced 3-fold, close to native PDO300 levels (Fig. 6). These data validate AmrZ as a negative regulator of *wzz2* promoter activity and therefore very long O antigen in mucoid P. aeruginosa. We also tested whether overexpression of *amrZ* in PAO1 *mucA22 amrZ*::Tn would complement this strain back to mucoid. When *amrZ* was induced, this strain became mucoid and alginate production was increased almost 50-fold (Table 2). Although there was a large increase in alginate produced when *amrZ* was induced, this was still 4 times less than the amount of alginate made by PDO300. This was surprising since the two strains looked similarly mucoid when grown on agar medium. As expected, overexpression of *amrZ* in PAO1 or PDO300 did not alter alginate production of these strains; PAO1 remained nonmucoid and PDO300 remained mucoid.

**TABLE 2:** Alginate produced by strains overexpressing *amrZ*

Strain	Inducer (1 mM IPTG)	Alginate (µg/ml)	SD	Fold change
PAO1 P_tac_-*amrZ*	−	BD^*a*^		
	+	BD		
PDO300 P_tac_-*amrZ*	−	184.75	23.10	
	+	187.28	37.76	1.01
PAO1 *mucA22 amrZ*::Tn P_tac_-*amrZ*	−	1.07	4.40	
	+	52.17	1.47	48.76

a BD, below detection.

### Disruption of *amrZ* restores Wzz2 production in mucoid strains.

**FIG 7:**
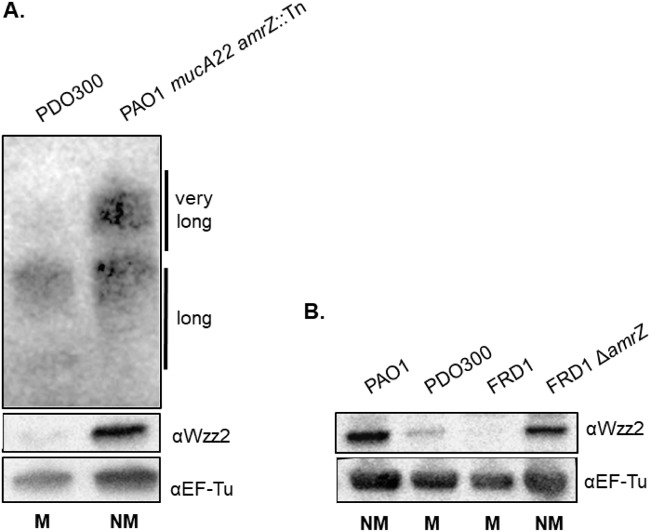
Disruption of *amrZ* restores Wzz2 production in mucoid strains. (A) Wzz2 and very long O antigen were monitored when *amrZ* was disrupted. (B) Mucoid LPS-rough clinical isolate FRD1 was originally isolated from a person with CF (39), and nonmucoid FRD1 Δ*amrZ* contains a clean deletion of *amrZ* (52). Western blotting was performed as described for Fig. 1. Abbreviations: NM, nonmucoid; M, mucoid.

We also wanted to confirm that disruption of *amrZ* in the context of *mucA22* would restore very long O antigen production. When protein and O antigen from PAO1 *mucA22 amrZ*::Tn were visualized using Western blot analysis, both Wzz2 and very long O antigen were restored to wild-type PAO1 levels (Fig. 7A). This supports our hypothesis that AmrZ also regulates very long O antigen production. To confirm that this regulation is dependent on *mucA22*, we measured O antigen in PAO1 *amrZ*::Tn. There is no difference in Wzz2 or very long O antigen when *amrZ* is disrupted in PAO1, which is expected since PAO1 does not characteristically produce high levels of *amrZ* (Fig. S2). This indicates that AmrZ does not regulate very long O antigen in the absence of *mucA* disruption. Finally, to support the finding that *amrZ* negatively regulates Wzz2 in our laboratory strains, we also wanted to determine if deletion of *amrZ* in a mucoid clinical isolates would restore Wzz2 production. Therefore, we tested Wzz2 levels in mucoid LPS-rough CF isolate FRD1 and an isogenic FRD1 *amrZ* mutant (23, 39, 45, 52). Comparably to other mucoid CF isolates, FRD1 does not produce detectable levels of Wzz2 (Fig. 7B). Deletion of *amrZ* in FRD1, however, greatly increases Wzz2 production levels. Expression of Wzz2 in the FRD1 *amrZ* mutant provides evidence that AmrZ inhibits Wzz2 expression in mucoid clinical isolates as well.

10.1128/mBio.02914-18.4 FIG S2 Disruption of *amrZ* in PAO1 does not alter Wzz2 or O antigen production. Wzz2 and serotype O5 antigen were monitored in each strain. PAO1 *amrZ*::Tn is a transposon mutant from the PAO1 library (1, 2). Samples were prepared and Western blotting was performed as described for Fig. 1. Abbreviations: NM, nonmucoid; M, mucoid. Download FIG S2, PDF file, 0.5 MB. Copyright © 2019 Cross and Goldberg. 2019 Cross and Goldberg This content is distributed under the terms of the Creative Commons Attribution 4.0 International license.

## DISCUSSION

P. aeruginosa isolates from chronic pulmonary infections exhibit unique characteristics, collectively termed the “chronic infection phenotype,” compared to isolates from other types of infections (11). These strains are typically mucoid and nonmotile and have an LPS-rough phenotype, defined as lacking the O antigen portion of LPS (11–15). Mucoid conversion is well studied (53, 54), as is the mechanism responsible for the nonmotile phenotype of chronic infection isolates (47, 55). On the other hand, little progress has been made in understanding how O antigen and the LPS-rough phenotype are regulated in the context of a chronic infection.

**FIG 8:**
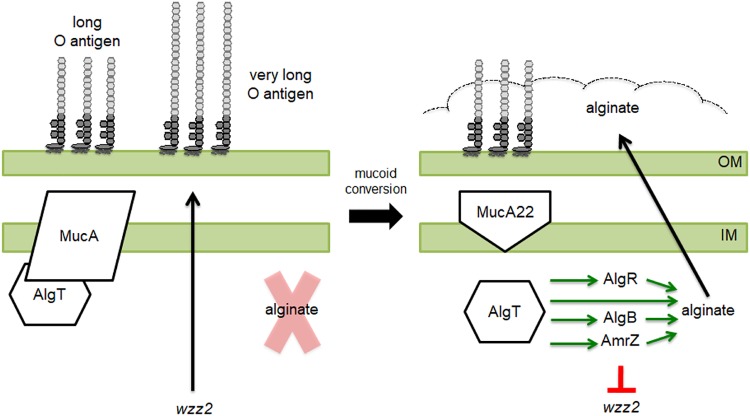
Model for the inverse regulation of alginate and very long O antigen in mucoid P. aeruginosa. (Left) In LPS-smooth nonmucoid P. aeruginosa, wild-type MucA sequesters the sigma factor AlgT, rendering it inactive. When AlgT is inactive, the AlgT regulon, including AmrZ and the alginate operon, is not transcribed. Therefore, *wzz2* and very long O antigen are expressed. (Right) When MucA acquires mutations, such as *mucA22*, AlgT is free to transcribe genes of its regulon. AmrZ now represses *wzz2*, resulting in loss of very long O antigen production. Abbreviations: IM; inner membrane, OM; outer membrane.

To fill this gap in knowledge, we first focused our studies on LPS-smooth mucoid laboratory strains in order to determine why O antigen production is altered in these strains and to identify possible regulators of O antigen chain length. In concentrating our studies on known transcription factors that are upregulated in mucoid strains, we found that AmrZ, which is an activator of alginate biosynthesis and a repressor of motility (47, 51, 55–57), negatively regulates *wzz2* promoter activity and therefore very long O antigen production (Fig. 6). From the data presented here, we can now build a model for the regulation of very long O antigen in mucoid P. aeruginosa (Fig. 8). Nonmucoid strains expressing wild-type *mucA* have little AlgT activity. Under these conditions, the *algT* regulon, including the alginate biosynthesis operon, *algB*, *algR*, and *amrZ*, are not transcribed. Because AmrZ is not produced, *wzz2* transcription is high and Wzz2 is free to mediate the assembly of very long O antigen. When *algT* expression is induced or *mucA* acquires mutations, AlgT is unrestricted and *amrZ* expression is induced. Overproduction of AmrZ represses *wzz2* expression and results in loss of very long O antigen chain lengths (Fig. 8).

While we are closer to understanding regulation of O antigen in mucoid P. aeruginosa, there may be other factors responsible for regulating chain length in other contexts. For example, McGroarty and Rivera reported that high temperatures, low pH, or low concentrations of phosphate or high concentrations of NaCl, MgCl_2_, glycerol, or sucrose resulted in decreased amounts of very long O antigen (58), but whether these conditions affect expression of *wzz2* has not been tested. Consequently, no known transcriptional regulators of O antigen chain length have been identified in nonmucoid P. aeruginosa. Conversely, temperature was shown to regulate transcription of the O antigen gene cluster in Yersinia enterocolitica (59). Additionally, the PmrA/B and Rcs systems were reported to directly regulate transcription of *wzz* genes in Salmonella enterica serovar Typhimurium (59, 60). Ongoing experiments in our laboratory aim to unravel additional circuits that regulate *wzz* expression in P. aeruginosa.

It is postulated that nonmucoid, mucoid, and nonmucoid revertant P. aeruginosa strains coexist during infection (61). In a recent study, 54% of nonmucoid CF isolates from 40 patients contained *mucA* mutations (62). Half of these also contained *algT* mutations, leading the authors to classify these strains as nonmucoid revertants. Surprisingly, the CFBRPA32 nonmucoid revertants and the PDO300 nonmucoid revertant we isolated each had an in-frame duplication of bp 127 to 135. Candido Caçador et al. also described a 9-bp insertion in this region (62). Sequence alignment of AlgT to Escherichia coli RpoE by Sautter et al., who also described mutations in this area, predicted that this region is involved in promoter melting (31). Mutations here may represent a hot spot for *algT* mutations that result in nonmucoid reversion.

Altogether, it appears that coordinating the overproduction of alginate with decreased Wzz2 and thereby very long O antigen is an important first step involved in establishing the chronic phenotype and may represent an intermediate phenotype between the transition from a nonmucoid LPS-smooth isolate to a mucoid LPS-rough isolate. Importantly, we have shown that different serotypes regulate very long O antigen through downregulation of Wzz2 (see Fig. S1 in the supplemental material) and that independent clinical isolates also downregulate Wzz2 (Fig. 1B, Fig. 4A, and Fig. 7B). Translating this across laboratory strains and CF isolates strengthens the relevance of our findings. Unlike *wzz1*, which is serotype specific and located at the beginning of the O antigen biosynthesis operon, *wzz2* is highly conserved among P. aeruginosa strains, and so having a regulatory circuit maintained within strains is not unexpected.

In support of this, nonmucoid strains of P. aeruginosa begin to express alginate during initial colonization of the lung (63), implying that *wzz2* and very long O antigen are also likely being repressed at this time. Loss of O antigen regulation was also observed for LPS-rough mucoid CF isolate 2192 (24). When the mutation responsible for the LPS-rough phenotype was identified and complemented back in *trans*, 2192 produced low-molecular-weight O antigen but not high-molecular-weight O antigen. This provides evidence that even mucoid LPS-smooth strains from CF still do not make very long O antigen. Still, these strains are usually LPS-rough, so why repress Wzz2? The benefit of regulating only Wzz2 and very long O antigen upon mucoid conversion, when the LPS-rough phenotype is ultimately selected, remains unexplained.

A long-term chronic P. aeruginosa infection model has not been established but will be crucial to determine the benefits of the coregulation of alginate and very long O antigen as well as the intermediate steps involved in the establishment of an LPS-rough phenotype. Understanding the coordinated regulation between the mucoid phenotype and O antigen expression will elucidate mechanisms that are selected for during the establishment of a long-term infection. Interfering with the expression of these chronic infection phenotypes and subsequent adaption of P. aeruginosa to the CF lung environment could provide a better outcome for people with CF.

## MATERIALS AND METHODS

### Bacterial strains and culture conditions.

Escherichia coli DH5α and SM10 were maintained on lysogeny broth (LB; Teknova) plates or in broth with or without 10 µg/ml tetracycline, 15 µg/ml gentamicin, 100 µg/ml carbenicillin, or 30 µg/ml kanamycin, as appropriate. P. aeruginosa strains were grown in LB medium or Vogel-Bonner minimal medium (VBMM) (64) supplemented with 100 µg/ml tetracycline, 60 µg/ml gentamicin, or 300 µg/ml carbenicillin, where necessary. For allelic exchange, 15% sucrose was used in no-salt LB plates (10 g/liter tryptone and 5 g/liter yeast extract). All plates were supplemented with 1.5% agar (Apex). Strains were grown at 37°C, and all conjugations were performed at 30°C as previously reported (65). Plasmids were transformed into electrocompetent PAO1 and PDO300, as previously described (66, 67). A complete list of strains and plasmids is available in Table S2, and a complete list of primers is available in Table S3. A detailed description of the construction of plasmids can be found in Text S1 in the supplemental material.

10.1128/mBio.02914-18.1 TEXT S1 Detailed description of materials and methods. Download Text S1, PDF file, 0.1 MB. Copyright © 2019 Cross and Goldberg. 2019 Cross and Goldberg This content is distributed under the terms of the Creative Commons Attribution 4.0 International license.

10.1128/mBio.02914-18.6 TABLE S2 Strains and plasmids used in this study. Download Table S2, PDF file, 0.1 MB. Copyright © 2019 Cross and Goldberg. 2019 Cross and Goldberg This content is distributed under the terms of the Creative Commons Attribution 4.0 International license.

10.1128/mBio.02914-18.7 TABLE S3 Primers used in this study. Download Table S3, PDF file, 0.1 MB. Copyright © 2019 Cross and Goldberg. 2019 Cross and Goldberg This content is distributed under the terms of the Creative Commons Attribution 4.0 International license.

### Mucoid to nonmucoid reversion.

CFBRPA32 and CFBRPA43 nonmucoid revertants were isolated as previously described (38). Passaging PDO300 in LB, without shaking, daily for 5 days isolated PDO300 nonmucoid revertant PDO300nmr1. Single-colony PCR using primers oAC221/oAC222 was used to amplify *algT*, which was then column purified (Qiagen miniprep kit) and sent to Genewiz for sequencing to identify *algT* mutations.

### Construction of PAO1 *mucA22 amrZ*::Tn and PA14 *mucA22*.

pEXG2-*mucA22* was transformed into chemically competent SM10 and then mated with PA14 or the PAO1 *amrZ*::Tn transposon mutant (68, 69) in a 3:1 ratio, according to the puddle-mating protocol described by Hmelo et al. (65). After patching, single-colony PCR using primers oAC089/oAC090 was used to amplify *mucA* from gentamicin-sensitive colonies. PCR-purified products were then sent to Genewiz for sequencing and verification that the *mucA22* allele had inserted into the strain.

### Sample preparation and Western blot analysis.

Exponential-phase bacterial cultures were normalized to an OD_600_ of 0.5 in 1 ml and centrifuged at 12,000 × *g* for 2 min. The cell pellet was resuspended in 50 μl of lysis solution (20 mM Tris, 1 mM EDTA, 10 mM MgCl_2_, 10 µg/ml DNase, 10 µg/ml RNase, and 1× GoldBio ProBlock protease inhibitor) and incubated at 37°C for 15 min. Fifty microliters of 2× Laemmli buffer (Bio-Rad) was added, the reaction mixture was boiled for 5 min, and LPS was prepared as previously reported (70), but without organic extraction by stopping after the proteinase K treatment. Proteins and LPS were separated by SDS-PAGE and transferred to a polyvinylidene difluoride (PVDF) membrane (Bio-Rad). The membranes were blocked in PBS-T containing 5% instant nonfat dry milk (Publix) and probed using specific polyclonal antibodies. Detailed sample preparation and Western blot analysis can be found in Text S1 in the supplemental material.

### qRT-PCR.

Quantitative reverse transcriptase PCR (qRT-PCR) was performed as previously described (16) with modifications described in Text S1 in the supplemental material.

### β-Galactosidase assay.

Reporter assays were performed as described previously by Miller (71) with modifications (72).

### Alginate isolation and quantification.

Alginate was purified and quantified as described previously (73) with modifications described in Text S1 in the supplemental material.

### Statistical analysis.

Data were analyzed using GraphPad Prism software (version 6). Experiments were compared using one-way analysis of variance (ANOVA) with Tukey’s multiple comparisons analysis or two-way ANOVA with Sidak’s multiple-comparison analysis. All data represent biological triplicate data with technical replicates. Graphs show mean values, and error bars represent standard deviation (SD). Significance is shown as follows: *, *P* < 0.05; **, *P* < 0.01; ***, *P* < 0.001; ****, *P* < 0.0001.

10.1128/mBio.02914-18.2 TEXT S2 Supplemental references. Download Text S2, PDF file, 0.09 MB. Copyright © 2019 Cross and Goldberg. 2019 Cross and Goldberg This content is distributed under the terms of the Creative Commons Attribution 4.0 International license.
