# Supplementary material for: Remodeling of O Antigen in Mucoid Pseudomonas aeruginosa via Transcriptional Repression of wzz2
Source: mBio. 2019 Feb 19;10(1):e02914-18. doi: 10.1128/mBio.02914-18 (PMC6381286; doi:10.1128/mBio.02914-18)
Supplement: FIG S1 [file mBio.02914-18-sf001.pdf]

**SUPPLEMENTAL MATERIAL**

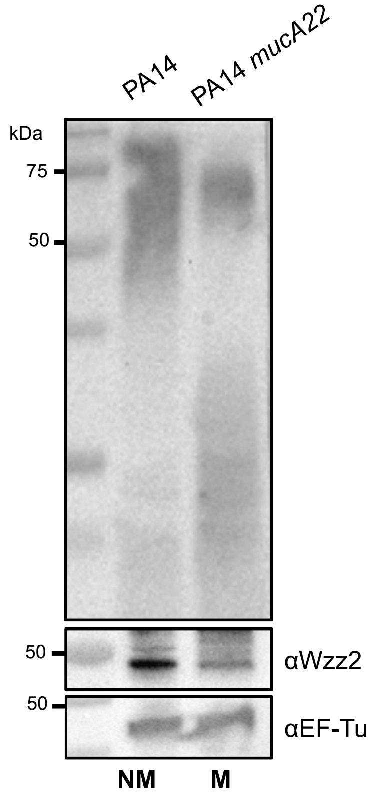


**Figure S1. Mucoid PA14 has reduced levels of Wzz2 and fewer very long O antigen chain-lengths compared to nonmucoid PA14.** Analysis of Wzz2 and serotype O10 antigen production. Samples were prepared and western blot was performed as described in Figure 1. Key: NM, nonmucoid; M, mucoid.
