# Supplementary material for: Remodeling of O Antigen in Mucoid Pseudomonas aeruginosa via Transcriptional Repression of wzz2
Source: mBio. 2019 Feb 19;10(1):e02914-18. doi: 10.1128/mBio.02914-18 (PMC6381286; doi:10.1128/mBio.02914-18)
Supplement: TABLE S1 [file mBio.02914-18-st001.pdf]

Table S1. Alginate produced by strains overexpressing *algT*

Strain	Inducer (1mM IPTG)	Alginate (µg/ml)	SD[a]	Fold Change
PDO300nmr1 P_{tac}-*algT*	-	29.48	19.02	
	+	26.52	21.40	0.90
PAO1 P_{tac}-*algT*	-	6.80	5.12	
	+	1101.15	76.90	161.85

[a]Standard Deviation
