# Supplementary material for: Remodeling of O Antigen in Mucoid Pseudomonas aeruginosa via Transcriptional Repression of wzz2
Source: mBio. 2019 Feb 19;10(1):e02914-18. doi: 10.1128/mBio.02914-18 (PMC6381286; doi:10.1128/mBio.02914-18)
Supplement: FIG S2 [file mBio.02914-18-sf002.pdf]

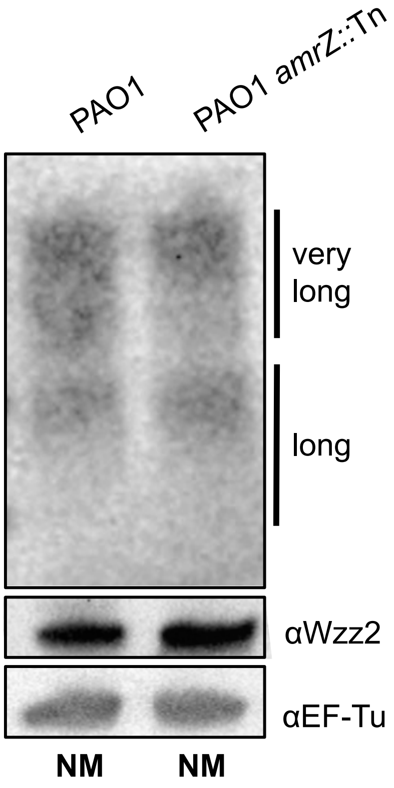

Figure S2. Disruption of *amrZ* in PAO1 does not alter Wzz2 or O antigen production. Wzz2 and serotype O5 antigen were monitored in each strain. PAO1 *amrZ*::Tn is a transposon mutant from the PAO1 library (1, 2). Samples were prepared and western blot was performed as described in Figure 1. Key: NM, nonmucoid; M, mucoid.
