# Supplementary material for: Remodeling of O Antigen in Mucoid Pseudomonas aeruginosa via Transcriptional Repression of wzz2
Source: mBio. 2019 Feb 19;10(1):e02914-18. doi: 10.1128/mBio.02914-18 (PMC6381286; doi:10.1128/mBio.02914-18)
Supplement: TEXT S1 [file mBio.02914-18-s0001.pdf]

Text S1.

Plasmid Construction. To generate pHERD20T-*wzz2*, the *wzz2* coding region was amplified using oAC017/oAC018 and the PCR product, along with pHERD20T, were digested using NcoI and HindIII (New England BioLabs). The *wzz2* fragment and digested pHERD20T were gel purified, ligated (New England BioLabs) in a 2:1 ratio, and the reaction was then transformed via heat-shock into chemically competent DH5α (Invitrogen) and selected for resistance on carbenicillin. Plasmids were PCR verified using oAC039/oAC040 and sequence confirmed.

Construction of miniCTX1-P_{wzz2}-optRBS-*lacZ* was accomplished by amplifying *lacZ* from miniCTX-*lacZ* using oJM524, which containing an consensus ribosome-binding site (taaggagg) (12), and oJM456. This PCR product, along with miniCTX1, was cut with PstI-HindIII (New England BioLabs) and ligated together to generate miniCTX1-optRBS-*lacZ*. Next, 500 bps of the upstream *wzz2* promoter region was amplified using oAC053/oAC054 and inserted into HindIII digest miniCTX1-optRBS-*lacZ* using isothermal assembly (Gibson Assembly Master Mix, New England BioLabs) following the manufacturers protocol. The reactions were transformed into chemically competent DH5α and selected for on the appropriate antibiotic. Plasmids were extracted from isolated colonies, PCR verified using oAC032/oAC033, and sequence verified. miniCTX1-P_{wzz2}-optRBS-*lacZ* was electroporated into electrocompetent PAO1, PDO300, PDO300nmr1, and PAO1 *mucA22 amrZ*::Tn as previously described (15, 17) to generate PAO1 attCTX::P_{wzz2}-optRBS-*lacZ*, PDO300 attCTX::P_{wzz2}-optRBS-*lacZ*, PDO300nmr1 attCTX::P_{wzz2}-optRBS-*lacZ*, and PAO1 *mucA22 amrZ*::Tn attCTX::P_{wzz2}-optRBS-*lacZ.* Insertions at the CTX site were verified using oAC056/oAC057.

In order to generate the inducible constructs miniTn*7*T-lacIq-Ptac-*amrZ* and miniTn*7*T-lacIq-Ptac-*algT* we amplified the coding sequence of *amrZ*, using oAC137/oAC138, or *algT,*

using oA221C/oAC222, from PAO1 using PCR. The amplified sequence was then inserted into HindIII digest miniTn*7*T-Gm-lacIq-P_{tac} using isothermal assembly (Gibson Assembly Master Mix, New England BioLabs) following the manufacturers protocol. All reactions were transformed into chemically competent DH5α and selected for on the appropriate antibiotic. Plasmids were extracted from isolated colonies, PCR verified using oAC148/oAC149, and sequence verified. miniTn*7*T-lacIq-Ptac-*amrZ* was electroporated into electrocompetent PAO1 attCTX::P_{wzz2}-optRBS-*lacZ,* PDO300 attCTX::P_{wzz2}-optRBS-*lacZ*, and PAO1 *mucA22 amrZ*::Tn attCTX::P_{wzz2}-optRBS-*lacZ* as previously described (15, 17) to generate PAO1 attCTX::P_{wzz2}-optRBS-*lacZ* attTn*7*::lacIq-Ptac-*amrZ,* PDO300 attCTX::P_{wzz2}-optRBS-*lacZ* attTn*7*::lacIq-Ptac-*amrZ*, and PAO1 *mucA22 amrZ*::Tn attCTX::P_{wzz2}-optRBS-*lacZ* attTn*7*::lacIq-Ptac-*amrZ.* miniTn*7*T-lacIq-Ptac-*algT* was electroporated into electrocompetent PAO1 attCTX::P_{wzz2}-optRBS-*lacZ* and PDO300nmr1 attCTX::P_{wzz2}-optRBS-*lacZ* to generate PAO1 attCTX::P_{wzz2}-optRBS-*lacZ* attTn7::lacIq-Ptac-*algT,* and PDO300nmr1 attCTX::P_{wzz2}-optRBS-*lacZ* attTn7::lacIq-Ptac-*algT*. 500 ng of pTNS3 helper plasmid was also included when transforming miniTn*7*T vectors. Insertions at the Tn*7* site were verified using oAC129/oAC130.

To generate pEXG2-*mucA22* the *mucA22* allele was PCR amplified from PDO300 using oAC089/oAC090 and inserted into HindIII digest pEXG2 using isothermal assembly (Gibson Assembly Master Mix, New England BioLabs) following the manufacturers protocol. The reactions were transformed into chemically competent DH5α and selected for on the appropriate antibiotic. Plasmids were extracted from isolated colonies, PCR verified using primers oAC091/oAC092, and sequence verified.

To overexpress *wzz2* in order to generate polyclonal antibody the *wzz2* coding region from nucleotides 150-1173 (to exclude transmembrane sequences in the first and last 150 bps of

the gene) was PCR amplified from *P. aeruginosa* strain PA103 using primers oJM483/oJM484 and cloned into pET28a digested with NdeI-XhoI (New England BioLabs) by isothermal assembly to construct pET28a-his6-*wzz2deltaTMS*. The Wzz2 N-terminus contains a 6X His-tag.

Generation of Wzz2 polyclonal antibodies. *E. coli* BL21(DE3) containing pET28a-his6-*wzz2deltaTMS* was induced with 0.2 mM IPTG at 16°C overnight in a 2.8 L Fernbach flask containing 1 L LB containing kanamycin. Cells were then lysed using a French press and protein was purified first by a nickel column and then by size exclusion (HiLoad16/60 Superdex200 FPLC). Purified Wzz2 was sent to and used by Covance (Princeton, NJ) to generate polyclonal antibodies in White New Zealand Rabbits.

Western blot analysis. Protein and LPS samples were thawed at room temperature, boiled for 5 minutes, and then cooled to room temperature. Proteins were separated by 10% SDS-PAGE and LPS was separated by 12% SDS-PAGE (Bio-Rad). Both were transferred to a polyvinylidene difluoride membrane (PVDF, Bio-Rad). The membranes were blocked in PBS-T containing 5% instant nonfat dry milk (Publix) and probed using specific polyclonal antibodies for O5 serotype antigen (1:2,500, Denka Seiken Co. Ltd. Group B), O10 serotype antigen (1:5,000 Denka Seiken Co. Ltd. Group H) Wzz2 (1:10,000), or a monoclonal antibody for EF-Tu (1:10,000, LS Bio) followed by a 1:10,000 dilution of horseradish peroxidase-conjugated anti-rabbit IgG (O5 antigen, O10 antigen, and Wzz2) or anti-mouse IgG (EF-Tu) immunoglobulin antibody (Sigma-Aldrich). Detection was performed using Pierce ECL Western Blotting Substrate (following manufacturer's instructions) and chemiluminescence detection. Images were obtained using a ChemiDoc Imaging System (Bio-Rad) and densitometry analyzed was performed using Image

Lab volume 5.2.1 software (Bio-Rad) relative quantity tool.

qRT-PCR. 500 µl of exponential phase culture was added to 1 ml of RNA Protect (Qiagen), incubated at room temperature for 5 minutes, and then centrifuged at 5,000 x g for 10 minutes. Pellets were air-dried and stored at -80°C. Total RNA was extracted using the MasterPure RNA Purification Kit (Epicentre) following the manufacturers protocol and 10 µg of total RNA was converted into cDNA using the SuperScript III First-Strand Synthesis System Kit (Invitrogen) following the manufacturer's instructions. Random hexamers were used to convert 1 µg of RNA to cDNA. Finally, cDNA was used to perform qPCR using *wzz2* specific primers oAC001/oAC002 and Fast Start Universal SybrGreen Master Mix (Roche) for detection. The amount of *wzz2* expressed was quantified based on cycle threshold (C_T) and primer efficiencies were determined using a standard curve of purified genomic DNA. All samples were then normalized to the housekeeping gene *rpoD* that was detected using oAC003/oAC004.

Alginate isolation and quantification. Single colonies were used to inoculate 3 ml LB and allowed to grow, rolling at 37°C, until turbid. This culture was back-diluted to an OD_{600} of 0.01 into 20 ml LB and allowed to grow overnight at 37°C, shaking. 10 ml of overnight culture was combined with 10 ml of 0.85% sodium chloride (Sigma) in a 50 ml conical tube, vortexed, and centrifuged at 12,000 x g for 30 minutes to remove cell material. The supernatant was transferred to a clean 50 ml conical tube and combined with 20 ml of 2% cetyl pyridinium (ACROS Organics), inverted 10 times to precipitate the alginate, and then centrifuged for at 12,000 x g for 10 minutes. The supernatant was carefully poured off of the pellet and discarded. The alginate pellet was resuspended in 10 ml of 1 M sodium chloride, vortexed thoroughly, a pipet was used

to break up the pellet, and then incubated at room temperature for 30 minutes to ensure pellet had gone into solution. 10 ml of isopropanol (Fisher Scientific) was added to the solution, inverted 10 times to reprecipitate the alginate, vortexed thoroughly, and then the solution was centrifuged again at 12,000 x g for 10 minutes. The supernatant was carefully poured off of the clear alginate pellet and a pipet was used to remove any residual isopropanol. Finally, the alginate pellet was resuspended in 10 ml 0.85% sodium chloride and a pipet was used to break up the pellet. Final suspension was incubated at 4°C overnight.

Alginate was quantified by heating at 55°C in a borate-carbazole solution. All values were compared to a standard curve generated by diluting laboratory grade alginic acid (Sigma) in 0.85% sodium chloride to concentrations of 100-900 ug/ml. Briefly, 6 µl of purified alginate or standard was added to 200 µl of 0.1 M H_3BO_3 reagent (created by adding 5 ml of 2 M BO_3^{-3} added to 95 ml of ACS- grade H_2SO_4). The 2 M BO_3^{-3} stock was created by adding 24.74g H_3BO_3 to 45 ml 4 M KOH, with heat, and then diluted to 200 ml in water. Next, 6 µl of 0.1% carbazole reagent (Sigma) was added and this solution was incubated at 55°C for 30 minutes in a water bath to allow for sufficient purple color to develop. After incubation, the solution was mixed and transferred to a 96-well plate. Absorbance was measured at 530 nm in a BioTek Synergy H1M plate reader and values were compared to the standard curve generated.
