# Supplementary material for: Remodeling of O Antigen in Mucoid Pseudomonas aeruginosa via Transcriptional Repression of wzz2
Source: mBio. 2019 Feb 19;10(1):e02914-18. doi: 10.1128/mBio.02914-18 (PMC6381286; doi:10.1128/mBio.02914-18)
Supplement: TABLE S2 [file mBio.02914-18-st002.pdf]

Table S2. Strains and plasmids used in this study.

Bacterial Strain or Plasmid	Description	Reference
E. coli		
DH5α	plasmid maintenance	Invitrogen
BL21(DE3)	protein expression and purification	Invitrogen
SM10	bi-parental mating	3
P. aeruginosa		
PAO1	nonmucoid laboratory strain, serotype O5	4
PAO1 *wzz2*::Tn	PAO1 transposon mutant PW2707	1,2
PAO1 *amrZ*::Tn	PAO1 transposon mutant PW6710	1,2
PDO300	mucoid PAO1 containing *mucA22* allelle	5
PDO300nmr1	nonmucoid revertant of PDO300, *algT*127-135 duplication	This study
PAO1 *mucA22 amrZ*::Tn	PAO1 transposon mutant PW6710 with *mucA22* allele	This study
PA14	nonmucoid laboratory strain, serotype O10	6
PA14 *mucA22*	mucoid PA14 containing *mucA22* allelle	This study
Clinical P. aeruginosa isolates		
CFBRPA10	cystic fibrosis nonmucoid clinical isolate	This study
CFBRPA34	cystic fibrosis nonmucoid clinical isolate	This study
CFBRPA44	cystic fibrosis nonmucoid clinical isolate	This study
CFBRPA20	cystic fibrosis nonmucoid clinical isolate	This study
CFBRPA08	cystic fibrosis nonmucoid clinical isolate	This study
CFBRPA11	cystic fibrosis mucoid clinical isolate	This study
CFBRPA33	cystic fibrosis mucoid clinical isolate	This study
CFBRPA32	cystic fibrosis mucoid clinical isolate	This study
CFBRPA32nmr1	nonmucoid revertant of CFBRPA32, *algT*127-135 duplication	This study
CFBRPA32nmr2	nonmucoid revertant of CFBRPA32, *algT*127-135 duplication	This study
CFBRPA43	cystic fibrosis mucoid clinical isolate	7
CFBRPA43nmr1	nonmucoid revertant of CFBRPA43, mutations not in *algT*	This study
CFBRPA43nmr2	nonmucoid revertant of CFBRPA43, *algT*C245A transversion	This study
FRD1	mucoid clinical CF isolate	8
FRD1 Δ*amrZ*	FRD1 with *amrZ* deleted	9
Plasmids		
miniCTX1	attCTX integration proficient	10
miniCTX1-*lacZ*	miniCTX1 containing promoterless-*lacZ* reporter	11
miniCTX1-optRBS-*lacZ*	miniCTX1 promoterless-*lacZ* reporter with optimized RBS	This study
miniCTX1-P*wzz2*-optRBS-*lacZ*	contains -25 to -500 bp of *wzz2* promoter	This study
pEXG2	allelelic exchange vector	12
pEXG2-*mucA22*	contains *mucA22* allele amplified from PDO300	This study
miniTn7T-Gm-lacIq-Ptac	attTn*7* integration proficient, IPTG inducible	13
miniTn7T-Gm-lacIq-Ptac-*algT*	contains coding region of *algT* for overexpression	This study
miniTn7T-Gm-lacIq-Ptac-*amrZ*	contains coding region of *amrZ* for overexpression	This study
pET28a	protein expression vector	Addgene
pET28a-his6-*wzz2*deltaTMS	expression vector containing His-tagged *wzz2* (without TMS)	This study
pHERD20T	multicopy overexpression plasmid, arabinose inducible	15
pHERD20T-*wzz2*	contains *wzz2* coding sequence for multicopy expression	This study
pHERD20T-*algT*	contains *algT* coding sequence for multicopy expression	15
pTNS3	transposase helper	16
