# Supplementary material for: Remodeling of O Antigen in Mucoid Pseudomonas aeruginosa via Transcriptional Repression of wzz2
Source: mBio. 2019 Feb 19;10(1):e02914-18. doi: 10.1128/mBio.02914-18 (PMC6381286; doi:10.1128/mBio.02914-18)
Supplement: TEXT S2 [file mBio.02914-18-s0002.pdf]

SUPPLEMENTAL REFERENCES

1. **Held K, Ramage E, Jacobs M, Gallagher L, Manoil C.** 2012. Sequence-verified two-allele transposon mutant library for Pseudomonas aeruginosa PAO1. J Bacteriol **194:**6387-6389.

2. **Jacobs MA, Alwood A, Thaipisuttikul I, Spencer D, Haugen E, Ernst S, Will O, Kaul R, Raymond C, Levy R, Chun-Rong L, Guenthner D, Bovee D, Olson MV, Manoil C.** 2003. Comprehensive transposon mutant library of Pseudomonas aeruginosa. Proc Natl Acad Sci U S A **100:**14339-14344.

3. **Simon R, Priefer U, Puhler A.** 1983. A Broad Host Range Mobilization System for in vivo Genetic Engineering: Transposon Mutagenesis in Gram Negative Bacteria. Biotechnology**:**784-791.

4. **Hancock REW, Carey AM.** 1979. Outer Membrane of Pseudomonas aeruginosa: Heat- and 2- Mercaptoethanol-ModifiableProteins. J Bacteriol **140:**902-910.

5. **Mathee K, Ciofu O, Sternbrg C, Lindum PW, Campbell JIA, Jensen P, Johnsten AH, Givskov M, Ohman DE, Molin S, Hoiby N, Kharazmi A.** 1999. Mucoid conversion of Pseudmonas aeruginosa by hydrogen peroxide: a mechanism for virulence activation in the cystic fibrosis lung. Microbiology **145:**1349-1357.

6. **Rahme LG, Stevens EJ, Wolfort SF, Jing Shao J, Tompkins RG, Ausubel FM.** 1995. Common Virulence Factors for Bacterial Pathogenicity in Plants and Animals. Science **268:**1899-1902.

7. **Limoli DH, Whitfield GB, Kitao T, Ivey ML, Davis Jr. MR, Grahl N, Hogan DA, Rahme LG, Howell PL, O'Toole GA, J.B G.** 2017. Pseudomonas aeruginosa Alginate Overproduction Promotes Coexistence with Staphylococcus aureus in a Model of Cystic Fibrosis Respiratory Infection. mBio **8**.

8. **Ohman DE, Chakrabarty AM.** 1981. Genetic Mapping of Chromosomal Determinants for the Production of the Exopolysaccharide Alginate in a Pseudomonas aeruginosa Cystic Fibrosis Isolate. Infect Immun **33:**142-148.

9. **Ramsey DM, Baynham PJ, Wozniak DJ.** 2005. Binding of Pseudomonas aeruginosa AlgZ to sites upstream of the algZ promoter leads to repression of transcription. J Bacteriol **187:**4430-4443.

10. **Hoang TT, Kutchma AJ, Becher A, Schweizer HP.** 2000. Integration-proficient plasmids for Pseudomonas aeruginosa: site-specific integration and use for engineering of reporter and expression strains. Plasmid **43:**59-72.

11. **Becher A, Schweizer H.** 2000. Integration-Proficient Pseudomonas aeruginosa Vectors for Isolation of Single-Copy Chromosomal lacZ and lux Gene Fusions. BioTechniques **29**.

12. **Rietsch A, Vallet-Gely I, Dove SL, Mekalanos JJ.** 2005. ExsE, a secreted regulator of type III secretion genes in Pseudomonas aeruginosa. Proc Natl Acad Sci U S A **102:**8006-8011.

13. **Meisner J, Goldberg JB.** 2016. The Escherichia coli rhaSR-PrhaBAD Inducible Promoter System Allows Tightly Controlled Gene Expression over a Wide Range in Pseudomonas aeruginosa. Appl Environ Microbiol **82:**6715-6727.

14. **Choi KH, Schweizer HP.** 2006. mini-Tn7 insertion in bacteria with single attTn7 sites: example Pseudomonas aeruginosa. Nat Protoc **1:**153-161.

15. **Qiu D, Damron FH, Mima T, Schweizer HP, Yu HD.** 2008. PBAD-based shuttle vectors for functional analysis of toxic and highly regulated genes in Pseudomonas and Burkholderia spp. and other bacteria. Appl Environ Microbiol **74:**7422-7426.

16. **Choi KH, Mima T, Casart Y, Rholl D, Kumar A, Beacham IR, Schweizer HP.** 2008. Genetic tools for select-agent-compliant manipulation of Burkholderia pseudomallei. Appl Environ Microbiol **74:**1064-1075.

17. **Savli H, Karadenizli A, Kolayli F, Gundes S, Ozbek U, Vahaboglu H.** 2003. Expression stability of six housekeeping genes: a proposal for resistance gene quantification studies of Pseudomonas aeruginosa by real-time quantitative RT-PCR. Journal of Medical Microbiology **52:**403-408.

18. **Choi KH, Kumar A, Schweizer HP.** 2006. A 10-min method for preparation of highly electrocompetent Pseudomonas aeruginosa cells: application for DNA fragment transfer between chromosomes and plasmid transformation. J Microbiol Methods **64:**391-397.
